# Galectin-3 Up-Regulation in Hypoxic and Nutrient Deprived Microenvironments Promotes Cell Survival

**DOI:** 10.1371/journal.pone.0111592

**Published:** 2014-11-04

**Authors:** Rafael Yamashita Ikemori, Camila Maria Longo Machado, Karina Mie Furuzawa, Suely Nonogaki, Eduardo Osinaga, Kazuo Umezawa, Marcelo Alex de Carvalho, Liana Verinaud, Roger Chammas

**Affiliations:** 1 Faculdade de Medicina da Universidade de São Paulo, Instituto do Câncer do Estado de São Paulo, São Paulo, SP, Brazil; 2 Laboratório de Investigação Médica em Medicina Nuclear – LIM43, São Paulo, SP, Brazil; 3 Departamento de Patologia do Instituto Adolfo Lutz, São Paulo, SP, Brazil; 4 Facultad de Medicina de La Universidad de La Republica, Montevideo, Uruguay; 5 Aichi-Medical University, Nagakute, Japan; 6 Instituto Federal do Rio de Janeiro, Instituto Nacional do Câncer, Rio de Janeiro, RJ, Brazil; 7 Departamento de Microbiologia e Imunologia, Instituto de Biologia, UNICAMP, Campinas, SP, Brazil; Wayne State University, United States of America

## Abstract

Galectin-3 (gal-3) is a β-galactoside binding protein related to many tumoral aspects, *e.g*. angiogenesis, cell growth and motility and resistance to cell death. Evidence has shown its upregulation upon hypoxia, a common feature in solid tumors such as glioblastoma multiformes (GBM). This tumor presents a unique feature described as pseudopalisading cells, which accumulate large amounts of gal-3. Tumor cells far from hypoxic/nutrient deprived areas express little, if any gal-3. Here, we have shown that the hybrid glioma cell line, NG97ht, recapitulates GBM growth forming gal-3 positive pseudopalisades even when cells are grafted subcutaneously in nude mice. *In vitro* experiments were performed exposing these cells to conditions mimicking tumor areas that display oxygen and nutrient deprivation. Results indicated that gal-3 transcription under hypoxic conditions requires previous protein synthesis and is triggered in a HIF-1α and NF-κB dependent manner. In addition, a significant proportion of cells die only when exposed simultaneously to hypoxia and nutrient deprivation and demonstrate ROS induction. Inhibition of gal-3 expression using siRNA led to protein knockdown followed by a 1.7–2.2 fold increase in cell death. Similar results were also found in a human GBM cell line, T98G. *In vivo*, U87MG gal-3 knockdown cells inoculated subcutaneously in nude mice demonstrated decreased tumor growth and increased time for tumor engraftment. These results indicate that gal-3 protected cells from cell death under hypoxia and nutrient deprivation *in vitro* and that gal-3 is a key factor in tumor growth and engraftment in hypoxic and nutrient-deprived microenvironments. Overexpression of gal-3, thus, is part of an adaptive program leading to tumor cell survival under these stressing conditions.

## Introduction

Galectins are a family of lectins with β-galactoside binding domains (carbohydrate recognition domains, CRDs). Fifteen galectins have been identified so far and divided into 3 subgroups: prototype, chimera and tandem. Gal-3 is the only galectin belonging to the chimera subgroup and it contains one CRD and an extended N-terminal domain [1]. It has a molecular mass ranging from 29 to 34 kDa and seems to be involved in increased cell motility [2], cell growth and angiogenesis [3]–[6], promoting cell resistance to reactive species of nitrogen and oxygen [7] and it is important in the formation of metastatic colonies [8].

Gal-3 plays different roles, occasionally in opposite ways, depending on its sub-cellular localization; (i) in the nucleus, it participates in the processing of pre-mRNA [9] and control of expression of selected genes [10], [11]; (ii) in the cytoplasm, it acts inhibiting apoptosis [12]–[14]; (iii) extracellularly, it acts as a deadhesion molecule interfering with cell-cell interactions [15], cell-matrix interactions [16], [17] and also participates in the induction of apoptosis [18]. And, at least in part, sub-cellular compartimentalization of gal-3 seems to be phosphorylation dependent [4], [19].

Some studies have demonstrated that gal-3 can be modulated by hypoxia, a common feature in solid tumors [20]–[22]. Hypoxia occurs when cells are deprived of oxygen due to vaso-occlusion or deficient angiogenesis, causing also nutrient deprivation and leading to tumor necrosis [23]. This is one of the hallmarks of *glioblastoma multiformes* (GBM), a common Central Nervous System (CNS) tumor, accompanied by the presence of pseudopalisades, described as hypercellular areas around necrotic tissue environments, which are likely composed of cells actively migrating out the hypoxic/necrotic foci [23]–[25].

These pseudopalisading cells are from 5 to 50% less proliferative and from 6 to 20 times more prone to apoptosis than adjacent cells. Some molecules are strongly involved in the biology of pseudopalisading cells, like the hypoxia inducible factor (HIF-1α) [24], [26] and gal-3, which is found expressed specifically within pseudopalisading cells [27] and has been widely studied in CNS tumors [28]–[32]. However, the roles of gal-3 in both oxygen *and* nutrient deprivation microenvironments are still unknown.

In this work, we analyzed the impact of hypoxia and serum deprivation on the expression pattern of gal-3 and its consequences in the survival of a hybrid human/murine glioma cell line, NG97ht [33], [34], and the human glioblastoma cell line, T98G *in vitro*. Also, we analyzed the *in vivo* impact of gal-3 knockdown in the tumor development of the human glioma U87MG cell line inoculated in nude mice. Here, we have shown that gal-3 expression is part of an adaptive program that protects glioma cells from death under hypoxia and serum deprivation and that it is also a key factor in the tumor growth and engraftment in ill perfused microenvironments, suggesting a protective role for gal-3 under these extreme stress conditions.

## Experimental Procedures

### Cell culture

The hybrid human/murine NG97ht glioblastoma cell line [33], [34] was cultured in RPMI 1640 medium containing 10–13% fetal bovine serum (FBS) and the human glioblastoma cell lines, U87MG (ATCC HTB-14) and T98G (ATCC CRL-1690), were cultured in DMEM low glucose containing 10% FBS. Cell cultures were incubated at 37°C in an atmosphere containing 95% air and 5% CO_2_.

### Xenotransplants derived from the NG97ht cell line

NG97ht xenotransplants were induced by subcutaneous inoculation of 1×10^6^ in the flank of nude mice. These animals were kept in sterile and specific pathogen free environments supplied with water and barren rations *ad libitum* for 20 days

Tumor tissues were harvested and formalin-fixed, dehydrated and paraffin embedded and then subjected to either routine eosin and hematoxylin staining or immunohistochemistry.

Procedures for immunohistochesmitry for gal-3 were performed as described previously by Neder *et al.* [27] using M3/38 rat anti-gal-3 monoclonal antibody (TIB166, from ATCC), where suitable anti-rat secondary antibodies conjugated with HRP were used. For the identification of hypoxic areas *in vivo*, we used the Hypoxyprobe-1 Kit (Chemicon International), as described by the manufacturer. For NF-κB immunohistochemistry, the SC-109 antibody (Santa Cruz) was used for the detection of the p65 subunit, using the Superpicture reagent (Life Technologies) as a secondary detection marker conjugated with HRP.

### Hypoxia and cobaltous chloride assays

Hypoxia was achieved by subjecting cells to an air flow composed by 95% of N_2_ and 5% of CO_2_ for 5 minutes within a Billups-Rothenberg – Modular Incubator Chamber (Del Mar, USA). After, the chamber was sealed. Partial pressure (p) of O_2_ was monitored using an oxygen sensor (PAC 3000, Dräeger, Germany) throughout the experiment and hypoxia was defined as pO_2_<1%.

Cobaltous chloride (CoCl_2_) was used as an agent capable of mimicking hypoxia by the stabilization of HIF-1α [35], [36] only for the NG97ht cell line in a concentration of 100 µM. Cells in normoxia were cultured under normal culture conditions as previously described.

### qRT-PCR for gal-3

NG97ht cells were cultured in triplicates in six-well plates and exposed to either normoxia, CoCl_2_ and hypoxia in 13% or 1% FBS for 3, 6, 24 and 48 h. Total RNA was extracted by TRIzol method and cDNA was obtained using SuperScript III enzyme (Invitrogen), as described by the manufacturer.

The qRT-PCR reactions were carried out with the following primers: murine gal-3 (sense-5′-CCT GGA GCT TAT CCT GGT CA -3′, anti-sense-5′-GTC ACC ACT GAT CCC CAG TTT-3′) and β-actin (sense-5′-AGA AAA TCT GGC ACC ACA CC-3′, anti-sense-5′-AGA GGC GTA CAG GGA TAG CA-3′). All reactions were performed in the Rotor Gene 3000 (Corbett Life Science) and analyzed using the Rotor Gene 6 software.

Gal-3 differential expression was determined by the Pfafll method of analysis [37], using β-actin as normalizer gene.

### Protein analysis

Cells were cultured and exposed to normoxia, CoCl_2_ (for NG97ht cells) and hypoxia in complete or deprived medium for 48 h. Cells were then washed with cold PBS and protein extractions were performed as described previously. Protein extracts were separated by 12% SDS-PAGE, using reducing sample buffers and the proteins were then electroblotted to PVDF membranes. The primary antibodies used were the M3/38 rat anti-gal-3, the B4-G2 mouse anti-caspase 7 (Biovision) and mouse anti-β-actin (SIGMA). Suitable secondary antibodies conjugated with peroxidase were then used.

### Two-dimensional gel electrophoresis

NG97ht and U87MG cells were exposed to normoxia, CoCl_2_ (NG97ht) and hypoxia in 1% FBS for 48 h. Cells were then washed with cold PBS and protein extractions were performed as described previously.

Protein extracts were treated with acid phosphatase 100 µg/mL in pipes buffer 40 mM pH6.0 for 30 minutes at 37°C and control treatment was performed using only pipes buffer. Proteins were precipitated in cold acetone three times their volume for 4 h at -20°C and they were centrifuged at 13000 rpm for 15 minutes at 4°C and then dried at room temperature.

Proteins were then subjected to the first dimension in Immobiline DryStrips 3–10 (Amersham), according to the manufacturer. Then, second dimension was performed in a 12% SDS-PAGE, electroblotted to PVDF and incubated in the M3/38 rat anti-gal-3 as previously described. Image analysis for the quantification of protein forms was performed using the ImageJ software.

### Translation inhibition with cycloheximide

NG97ht cells were cultured in quadruplicates in six-well plates and exposed to normoxia, CoCl_2_ or hypoxia in 1% FBS and to DMSO, cycloheximide (CHX) 5 µM or 20 µM for 6 h. Then, cells had their total RNA extracted and cDNA produced. qRT-PCR was performed and primers used were for the murine gal-3 sequence, as previously described, and murine RPLP0 (sense-5′-TGC CAC ACT CCA TCA TCA AT-3′, anti-sense-5′-CGA AGA GAC CGA ATC CCA TA-3′), used as normalizer gene.

### HIF inhibition with CAS 934593-90-5 and NF-κB inhibition with dehydroxymethylepoxyquinomicin (DHMEQ)

NG97ht cells were cultured in triplicates in six-well plates and exposed to either normoxia, CoCl_2_ or hypoxia in 1% FBS for 24 h and exposed to DMSO, the HIF-1α inhibitor 10 µM (CAS 934593-90-5, Calbiochem) or DHMEQ 5 µg/mL, an NF-κB inhibitor [38]. Then, total RNA was extracted and cDNA obtained and analyzed as previously described. VEGF and CCL5 gene induction was used as a control for the HIF-1α and NF-κB inhibition, respectively. Also protein extracts were performed and analyzed as previously described for the gal-3 detection.

### Immunofluorescence for HIF-1α

NG97ht cells were cultured in 13 mm coverslips and exposed to normoxia, CoCl_2_ and hypoxia in serum-deprived medium for 48 h, then fixed in 1% paraformaldehyde and processed for the detection of HIF-1α. Primary antibody used was the rabbit anti-HIF-1α GTX30124 (Genetex) and the secondary antibody was the anti-rabbit Alexa 488 (Invitrogen). Cells nuclei were stained with DAPI and analyzed in confocal microscopy (Zeiss LSM 510 Meta/UV).

### DNA content analysis by propidium iodide staining

NG97ht cells were cultured in triplicates in six-well plates, exposed to normoxia, CoCl_2_ or hypoxia in complete or deprived medium for 24, 48 and 72 h. For the evaluation of cell death, cells were analyzed for their DNA content by propidium iodide staining. Cells were harvested by trypsinization, fixed in ethanol 70% and analyzed by flow cytometry (FACScalibur) through the binding of propidium iodide [39], [40]. Briefly, cells were washed twice in PBS after fixation and were incubated in 20 µg/mL of propidium iodide, 200 µg/mL of RNAse and 0,1% v:v of Triton X-100 in PBS for 30 minutes protected from light, then cell death was analyzed by the evaluation of cellular DNA content by flow cytometry (FACScalibur BD), and subsequent identification of hypodiploid cells.

### Detection of reactive oxygen species (ROS) by diihydroethidium (DHE)

NG97ht cells were cultured in triplicates in six-well plates, exposed to normoxia, CoCl_2_ or hypoxia in complete or deprived medium for 48 h. For ROS detection, cells were incubated for 30 minutes at 37°C with DHE 5 µM diluted in medium and then harvested by trypsinization. Cells were centrifuged for 2 mins at 2000 rpm and ressuspended in PBS and the evaluation of ROS was performed by flow cytometry (FACScalibur BD) using the geometric mean of the median fluorescence intensity.

### Gal-3 specific siRNA and shRNA knockdown

For gal-3 siRNA knockdown assays, NG97ht cells were cultured in triplicates in six-well plates. Cells were washed with OPTIMEM medium (Gibco, Invitrogen) and then incubated with 100 pmols of gal-3 siRNA (5′UUU CCU GAU UAG UGC UCC ACC CGC CGC-3′; 5′-GGG GGG UGG AGC ACU AAU CAG GAA A-3′) or 100 pmols of scramble siRNA with 4 µl of Lipofectamine 2000 (Invitrogen). Oligonucleotides for gal-3 silencing and scramble oligonucleotides were purchased from IDT- Integrated DNA Technologies (Coralville, IA). Cells were kept for 6 h in an atmosphere with 37°C and 5% of CO_2_ in absence of FBS. Cells were then exposed to normoxia, CoCl_2_ or hypoxia in 13% or 1% FBS, as previously described, for 42 h. After this exposure, gal-3 mRNA and proteins were extracted and analyzed or cells were subjected to cell death analysis as previously described.

For T98G and U87MG gal-3 shRNA knockdown, cells were transduced with a lentivirus containing gal-3 shRNA sequence (OpenBiosystems, pLKO-shGAL3 Cat. TRC0000029305) and then subjected to cell selection by puromicyn (1 µg/mL for T98G and 0.5 µg/mL for U87MG). The shRNA scramble sequence was used as a transduction control. Gal-3 knockdown was evaluated by western blot and by qRT-PCR using primers for the β-actin and for the human gal-3 (sense-5′ -AGA CAG TCG GTT TTC CCA TTT -3′, anti-sense-5′ -ACC AGA AAT TCC CAG TTT GCT-3′). For T98G cell death analysis, cells were plated in triplicates in six-well plates and treated for 48 h in 10% or 1% FBS in either normoxia or hypoxia and then the DNA content was analyzed by flow cytometry (FACScalibur BD).

For the U87MG *in vivo* assay, 5×10^6^ cells were inoculated in the flank of nude mice using the scramble shRNA (n = 6) and gal-3 shRNA cells (n = 5). These animals were kept under sanitary barrier conditions and supplied with water and barren rations *ad libitum* until they had formed a tumor mass around 1 to 1.4 cm^3^.

Tumor sizes were measured every two to three days and were harvested, formalin-fixed, dehydrated and paraffin embedded and then subjected to routine eosin and hematoxylin staining.

### U87MG cell growth assay

U87MG cells transduced with scramble or gal-3 shRNA were analyzed for cell proliferation *in vitro*. Cells were cultured in triplicates in six-well plates (1×10^5^ per well) and counted for every 24 h after the first 48 h for six days for comparison between these two cells.

### Animal ethical committee approval

This work was approved by the Ethical Committee for Animal Experimentation (CEEA) (project number 0993/09, registry number 4673) and followed the guidelines of the Brazilian Council on Animal Care (COBEA).

### Statistical analysis

All statistical analysis was performed with the GraphPad Prism 5.0 software. Differences were considered statistically significant if the probability value was less than 0.05 and all data are presented as mean±SEM.

## Results

### NG97ht cell line recapitulates glioblastoma growth in nude mice and demonstrates accumulation of gal-3 within pseudopalisades

**Figure 1:**
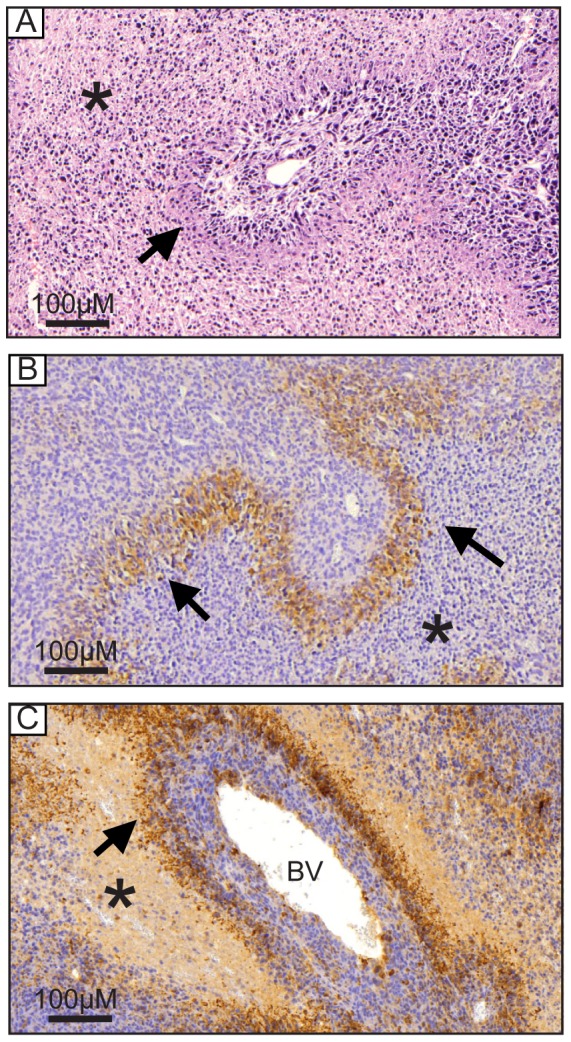
*In vivo* analysis shows hypoxia and gal-3 accumulation in pseudopalisades. NG97ht cells were transplanted subcutaneously in nude mice and tumors formed were excised and analyzed microscopically. ***A***. Tumors present necrotic foci (* all figures) and pseudopalisades (arrows). HE staining. ***B***. Tumors demonstrated hypoxic regions (pimonidazole adducts) in pseudopalisades (brown areas, arrows). ***C***. Immunohistochemistry for gal-3 demonstrated pseudopalisades intensely stained (brown areas, arrows). Sparse cells in non-necrotic areas around blood vessels (BV) were also positive. Scale bar 100 µm.

Based on histopathological analysis of tumor tissues, we could observe both necrosis (Fig. 1*) and the organization of pseudopalisades (Fig. 1A, arrows), as described by Grippo and coworkers [33]. Furthermore, we showed that pseudopalisading areas are hypoxic (Fig. 1B, arrows) due to staining of pimonidazole, and display an intense staining for gal-3 in the cytoplasm of pseudopalisading cells (Fig. 1C, arrows) and in scattered cells within the tumor. Gal-3 ligands were also detected in pseudopalisades (Figure S1).

### NG97ht cells in hypoxia and nutrient deprivation induce gal-3 expression and present multiple electrophoretic forms in vitro

Oxygen deprivation was achieved by culturing cells in hypoxic chambers (Figure S2), or alternatively mimicked through the exposure of cells to CoCl_2_, an inhibitor of prolyl-hydroxylases [35], [36].

**Figure 2:**
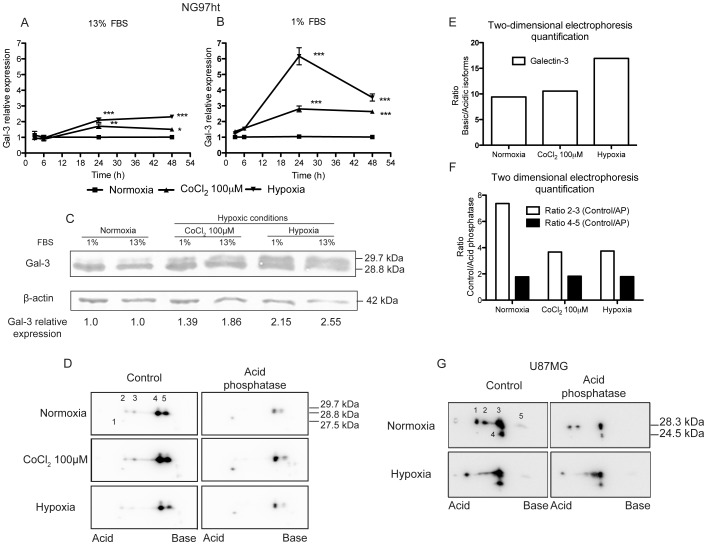
Gal-3 is upregulated in hypoxia in NG97ht cells. Analysis of gal-3 by qRT-PCR and western blot in NG97ht cells exposed to normoxia, CoCl_2_ and hypoxia in complete or serum-deprived medium. ***A/B***. qRT-PCR demonstrated gal-3 mRNA upregulation after 24 h of exposure either in complete (***A***) or serum-deprived medium (***B***) in cells exposed to CoCl_2_ and hypoxia compared to normoxia. β-actin was used as normalizer. Representative experiment performed at least in three independent assays and data are presented as mean±SEM. *p<0.05; **p<0.01; ***p<0.001. ***C***. Gal-3 protein accumulated after 48 h, in CoCl_2_ and hypoxia both in complete and serum-deprived medium. Two electrophoretic forms of gal-3, 28.8 kDa and 29.7 kDa, were observed. ***D***. NG97ht cell protein extracts derived from cells exposed to normoxia, CoCl_2_ and hypoxia in deprived medium for 48 h were submitted to treatment with acid phosphatase (AP) 100 µg/mL and analyzed by two-dimensional gel electrophoresis. Five main forms (1 to 5) were detected in the NG97ht cell line with their isoelectric point (IP) ranging from 8.00 to 9.33 and molecular sizes from 27.5 and 29.7 kDa. Spots 4 and 5 display each two forms of same IP, but different molecular sizes. ***E***. The quantification between isoforms 4–5 (basic) and 2–3 (acidic) demonstrated increase in the first mainly in hypoxia when compared to normoxia. ***F***. In the treatment of the protein extracts with AP, there was a decrease in isoforms 2 and 3 compared to control samples mainly in cells exposed to normoxia. Isoforms 4–5 did not demonstrate changes between normoxia, CoCl_2_ and hypoxia. ***G***. U87MG protein extracts derived from cells exposed to normoxia and hypoxia in deprived medium for 48 h also demonstrated differences in two-dimensional gel electrophoresis, showing five forms (1 to 5) with IP ranging from 7 to 9 and molecular sizes from 28.3 to 24.5 kDa. Treatment of protein extracts with AP resulted in the IP modification in gal-3 in normoxia.

**Figure 3:**
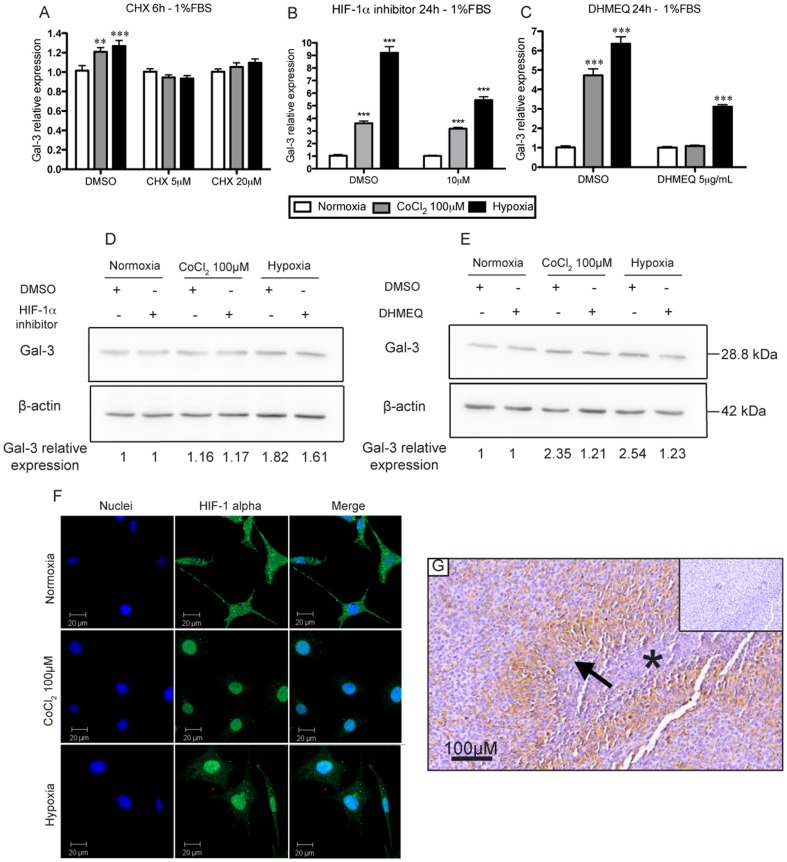
Gal-3 upregulation in hypoxia depends on HIF-1α and NF-κB and is blocked by cicloheximide. ***A***. qRT-PCR analysis of NG97ht cells treated with cycloheximide (CHX) 5 µM and 20 µM and its control (DMSO) and in cells exposed to either normoxia, CoCl_2_ or hypoxia in serum-deprived medium for 6 h demonstrated significant increase in gal-3 expression in cells exposed to CoCl_2_ and hypoxia compared to normoxia in the group treated with DMSO, but not when cells were exposed to CHX. ***B***. The exposure to HIF-1α inhibitor 10 µm (CAS 934593-90-5) demonstrated gal-3 decreased induction by qRT-PCR in cells exposed to hypoxia, but not normoxia and CoCl_2_. DMSO used as control. ***C***. The exposure to DHMEQ 5 µg/mL, an NF-κB inhibitor, decreased gal-3 induction by qRT-PCR both in CoCl_2_ and hypoxia compared to normoxia after 24 h in serum-deprived medium. DMSO used as control. ***D***. Western blot analysis with the HIF-1αinhibitor demonstrated a slight decrease in gal-3 only in cells exposed to hypoxia, but not normoxia and CoCl_2_, compared to DMSO control (experiment performed once). ***E***. Western blot analysis also demonstrated decreased gal-3 induction in cells treated with DHMEQ compared to DMSO in hypoxia and CoCl_2_ compared to normoxia after 24 h in serum-deprived medium. **F**. Cells in normoxia display scattered HIF-1α (green) throughout cytoplasm, while cells exposed to CoCl_2_ and hypoxia display HIF-1α mainly in cells nuclei (blue). ***G***. *In* vivo, NF-κB demonstrates to be upregulated in pseudopalisading areas, which display increased gal-3 expression. Representative experiments performed at least in three independent assays and data are presented as mean±SEM. **p<0,01; ***p<0.001.

It was observed gal-3 mRNA upregulation after 24–48 h either in complete or serum-deprived medium (Fig. 2A/B) in the NG97ht cell line. This hybrid cell line only expresses the murine gal-3 from its genome, since it lacks the human gal-3 gene. Protein analysis demonstrated accumulation of gal-3 in cells exposed to either CoCl_2_ or hypoxia after 48 h compared to normoxia (Fig. 3C). Two electrophoretic forms of gal-3, ranging from 28.8 kDa to 29.7 kDa, were observed in the experiments, with accumulation of the higher molecular weight form of gal-3.

Two-dimensional electrophoresis demonstrated five main gal-3 electrophoretic isoforms (Fig. 2D), 1 to 5 according to their isoelectric point (IP), ranging from 8.00 to 9.33. Protein extracts treated with acid phosphatase (AP) demonstrated decrease in forms 2 and 3. Quantification of these different gal-3 isoforms with the Image J software demonstrated that in hypoxia, basic isoforms were relatively increased compared to acidic isoforms (Fig. 2E) and that the treatment with AP decreased acidic isoforms in normoxia compared to hypoxia, but basic isoforms displayed the same rates of modifications in all conditions (Fig. 2F).

Also, mass spectrometry analysis of gal-3 derived from NG97ht showed two gal-3 variants expressed in this cell line, differing in the C-terminal domain of the protein (^251^Gly and ^251^Arg) explaining the sister isoforms 4 and 5 (data not shown). In addition, two-dimensional electrophoresis of protein extracts from the U87MG cell line demonstrated IP variations ranging from 7 to 9. In normoxia, gal-3 displayed acid phosphatase-sensitive modifications, which were less detected in cells exposed to hypoxia (Fig. 2G). Altogether, these results indicated that, if any alterations are observed, gal-3 seems less phosphorylated under hypoxic conditions.

### Gal-3 expression depends on de novo protein synthesis and on HIF-1α and NF-κB in vitro when exposed to hypoxia and nutrient deprivation

To evaluate whether gal-3 induction under CoCl_2_ and hypoxia was due to factors already present in NG97ht cells prior to the exposure to hypoxia, we treated these cells with cycloheximide, an inhibitor of protein translation, and verified if gal-3 upregulation was resistant to it. As observed in Fig. 3A, upon exposure to cycloheximide, no gal-3 upregulation was observed in serum-deprived cells in either CoCl_2_ or hypoxia when compared to cells exposed to DMSO. We further evaluated the dependence of both HIF-1α [41]–[44] and NF-κB [45] in nutrient and oxygen deprived dependent gal-3 induction.

The HIF-1α inhibitor (compound CAS 934593-90-5), which inhibits HIF-1α accumulation and gene transcription activity, led to a decrease in gal-3 induction in cells exposed to hypoxia and nutrient deprivation both by mRNA (Fig. 3B) and protein analysis (Fig. 3D). The immunofluorescence for HIF-1α in NG97ht cells demonstrated that this protein is not accumulated in the nucleus in cells in normoxia, but only when cells are exposed to CoCl_2_ and hypoxia (Fig. 3F).

To evaluate the NF-κB putative roles, we exposed NG97ht cells to dehydroxymethylepoxyquinomicin (DHMEQ), which inhibits NF-κB translocation to the nucleus and DNA binding, or DMSO (as control) in normoxia, CoCl_2_ and hypoxia in serum-deprived medium. Our results demonstrated that DHMEQ decreased gal-3 induction under hypoxia exposure when compared to DMSO both in mRNA (Fig. 3C) and protein levels (Fig. 3E).

Immunohistochemistry for NF-κB *in vivo* in the tumor mass originated from the inoculation of NG97ht cells in the flank of nude mice demonstrated its accumulation in pseudopalisading cells (Fig. 3G, arrows) both in the cytoplasm and in the cells nuclei.

### Combined oxygen and serum deprivation, but not hypoxia alone, trigger NG97ht cell death, which is accompanied by the induction of ROS

**Figure 4:**
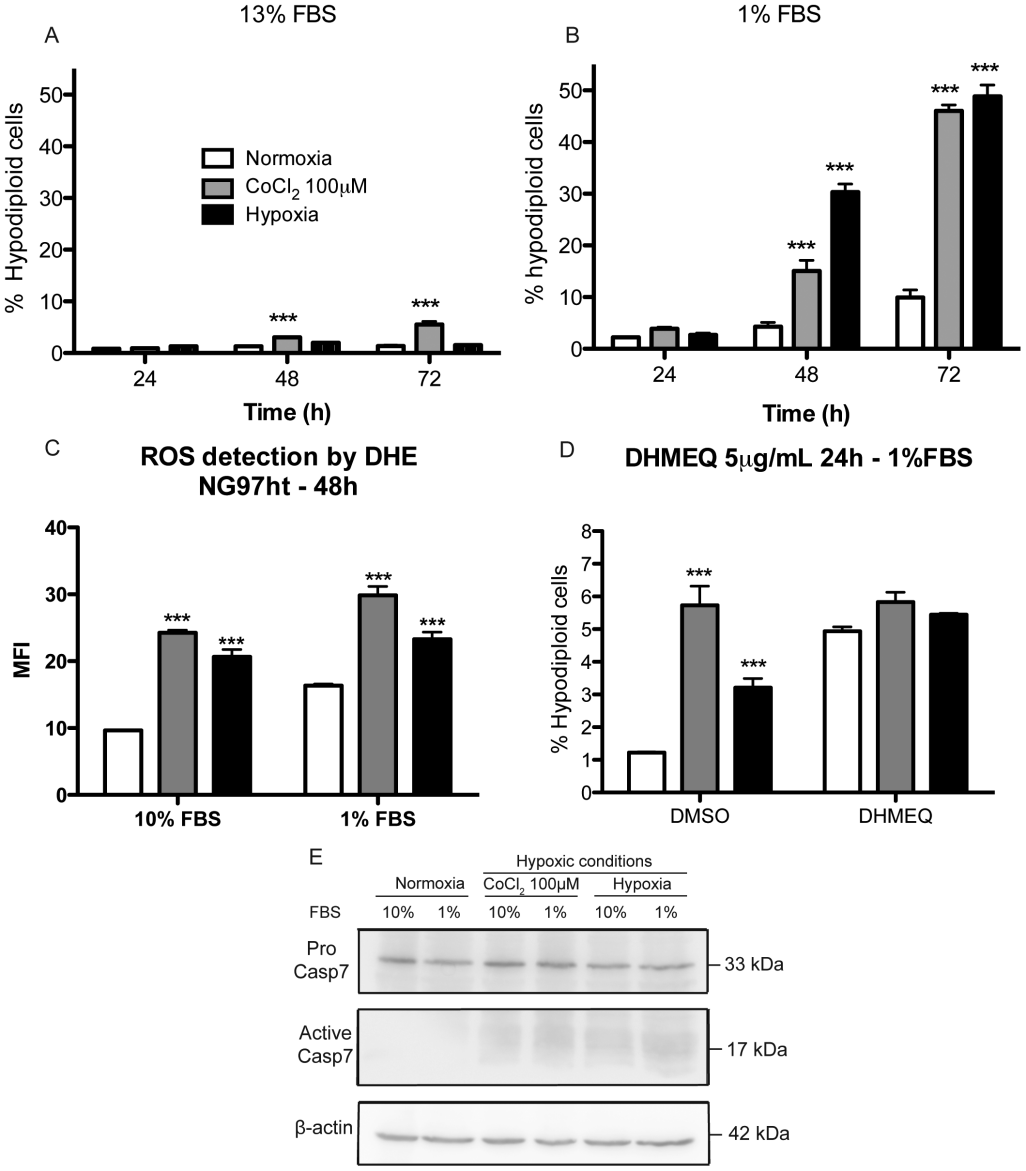
NG97ht cells demonstrate increased cell death only when exposed to oxygen and nutrient deprivation, which is accompanied by the increase of reactive oxygen species and caspase-7 activation. *A*. Experiments performed in complete medium did not demonstrate differences in cell death rates in cells exposed to hypoxia compared to normoxia, however, cells in CoCl_2_ demonstrated cell death after 48 h of exposure. *B*. In serum-deprived medium, longer periods of exposure (>24 h) significant cell death rates were observed in CoCl_2_ and hypoxia compared to normoxia. *C*. ROS detection by DHE also demonstrated the increase of these molecules in cells exposed to hypoxia and CoCl_2_ regardless serum concentrations and in normoxia in nutrient deprivation compared to normoxia in complete medium. *D*. NG97ht cells exposed to DHMEQ in serum-deprived medium presented increased cell death in cells exposed to normoxia and hypoxia, but not CoCl_2_. *E*. Caspase 7 analysis showed increased active caspase-7 in cells exposed do CoCl_2_ and hypoxia, both in complete or serum-deprived medium. Representative experiments performed at least in three independent assays and data are presented as mean±SEM. *p<0.05; **p<0.01; *** p<0.001.

To evaluate the cell death rates at different experimental conditions, cell death assays with propidium iodide staining were performed. In complete medium, cell death rates were the same in both normoxic and hypoxic conditions (Fig. 4A), however, when cells were exposed to oxygen and serum deprivation, increased cell death rates were observed after 48–72 h (Fig. 4B). Regarding cells exposed to CoCl_2_, we could observe increased cytotoxicity, regardless serum concentration, in time points >24 h (Fig. 4A/B). The detection of reactive oxygen species (ROS) (Fig. 4C) demonstrated ROS induction in cells exposed to CoCl_2_ and hypoxia both in complete or deprived medium.

Also, our studies demonstrated that DHMEQ increased cell death rates in cells exposed to both normoxia and hypoxia in nutrient deprivation (Fig. 4D) compared to controls exposed to DMSO.

Caspase 7 analysis by western blot assays demonstrated increased active caspase 7 in cells exposed to CoCl_2_ and hypoxia compared to normoxia both in complete or serum-deprived medium (Fig. 4D).

### Gal-3 knockdown increases cell death in oxygen and serum deprived cells

**Figure 5:**
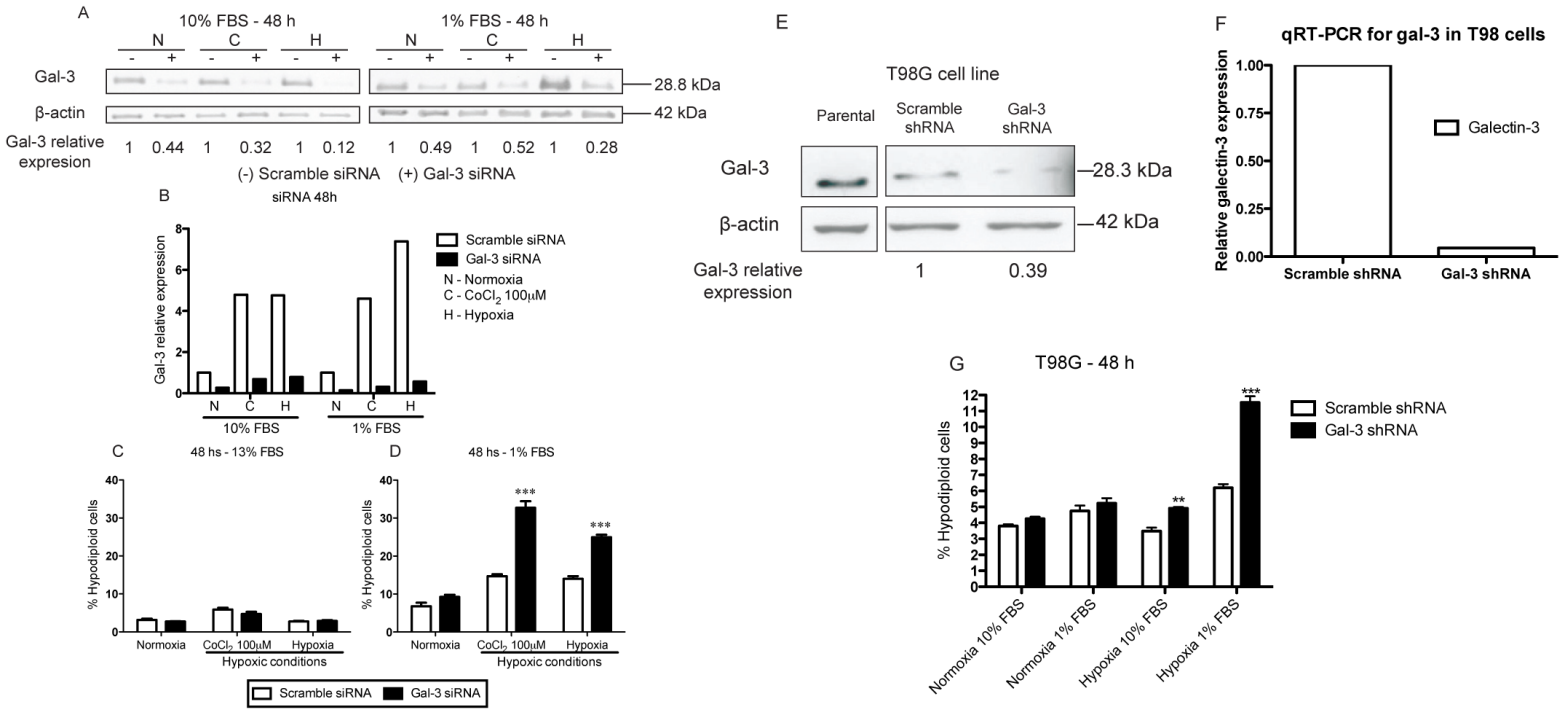
Gal-3 knockdown sensitizes cells to cell death in oxygen and nutrient deprivation in the NG97ht cell line. Analysis of NG97ht cells incubated with specific gal-3 siRNA or scramble siRNA for 6 h and then exposed to either normoxia (N), CoCl_2_ (C) or hypoxia (H) in complete or serum-deprived medium for additional 42 h. ***A***. Protein analysis demonstrated gal-3 knockdown in each experimental condition. Gal-3 knockdown was calculated relatively to its β-actin control. ***B***. In addition, gal-3 mRNA was evaluated and qRT-PCR demonstrated significant knockdown upon treatment with specific siRNA compared to scramble siRNA. ***C***. Analysis of hypodiploid cells demonstrated no differences in cell death rates in cells exposed to high serum concentrations, regardless the levels of gal-3 expression. ***D***. Upon serum-deprivation, cells were sensitized to cell death in hypoxic conditions. Sensitization was much more pronounced upon knockdown of gal-3, as compared to cells transfected with scramble siRNA. T98G cells transduced with gal-3 shRNA demonstrated decreased gal-3 protein by western blot (***E***) and mRNA by qRT-PCR (***F***) compared to scramble shRNA. Gal-3 knockdown was calculated relatively to its β-actin control. ***F***. Analysis of hypodiploid cells demonstrated increased cell death in cells transduced with gal-3 shRNA compared to cells transduced with scramble shRNA when exposed both to hypoxia or hypoxia and serum-deprivation. Representative experiment performed at least in three independent assays and data are presented as mean±SEM. **p<0.01, ***p<0.001.

As shown in Fig. 5, incubation of NG97ht cells with specific gal-3 siRNA led to a significant decrease on gal-3 both in protein (Fig. 5A) and mRNA (Fig. 5B) levels. Inhibition of gal-3 gene expression led to no differences in cell death rates in cells exposed to complete medium (Fig. 5C) under either normoxic or hypoxic conditions. However, sensitization to cell death under exposure to hypoxia and serum deprivation conditions is the functional consequence of gal-3 inhibition, as shown in Fig. 5D. In addition, gal-3 knockdown led to a significant accumulation of cells in non-proliferating phases of the cell cycle in these treatments (Figure S3). These findings were also observed in another human glioblastoma cell line. In the T98G cell line, gal-3 was decreased by shRNA knockdown both in protein (Fig. 5E) and mRNA (Fig. 5F) levels and it was demonstrated increased cell death in cells with gal-3 knockdown compared to control cells in hypoxia and serum deprivation (Fig. 5G). Taken together, these data indicate a protective role of gal-3 under extreme stress conditions such as hypoxia and serum deprivation.

### U87MG gal-3 knockdown cells display decreased tumor growth and increased time for tumor engraftment in vivo

**Figure 6:**
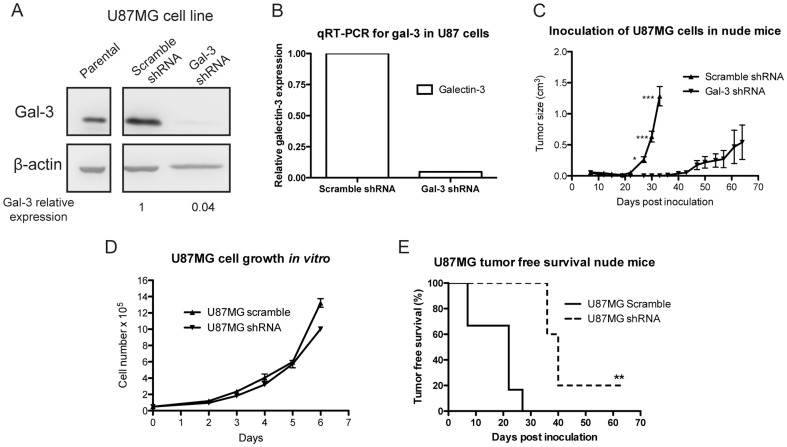
U87MG glioma cells transduced with gal-3 shRNA demonstrate decreased tumor size and growth. U87MG cells transduced with gal-3 shRNA demonstrated decreased gal-3 protein by western blot (***A***) and mRNA by qRT-PCR (***B***) compared to scramble shRNA. Gal-3 knockdown was calculated relatively to its β-actin control. ***C***. The inoculation of U87MG gal-3 shRNA cells in the flank of nuce mice (n = 5) demonstrated a decrease in tumor size and growth compared to the U87MG scramble shRNA (n = 6). ***D***. The delay presented for the decrease in tumor size and growth in the gal-3 shRNA cells was not due to a decreased tumor cell proliferation *in vitro* when compared to scramble shRNA cells. ***E***. Gal-3 knockdown cells demonstrated increased tumor free survival time compared to scramble shRNA cells. Representative experiments performed at least in three independent assays and data are presented as mean±SEM. *p<0.05, **p<0.01, ***p<0.001.

To analyze gal-3 possible roles *in vivo*, the human glioma cell line U87MG was subjected to gal-3 knockdown by shRNA and compared to its control. Results demonstrated both gal-3 protein and mRNA knockdown in U87MG cells transduced with gal-3 shRNA compared to control (Fig. 6A and B). *In vivo* assays inoculating these cells in the flank of nude mice displayed decreased size and growth rates (Fig. 6C) in tumors derived from the gal-3 shRNA cells compared to the scramble shRNA. These differences in tumor cell proliferation were not due to differences in cell proliferation *in vitro* since these cells demonstrated similar proliferation rates (Fig. 6D). Also, the analysis of the tumor engraftment rates demonstrated that gal-3 shRNA derived tumors presented increased delay compared to scramble shRNA (Fig. 6E).

## Discussion

In a previous work, we have demonstrated that gal-3 accumulated in glioma's pseudopalisades [27], which was also demonstrated by our results in the hybrid glioma cell line NG97ht. Concomitant with gal-3 accumulation in pseudopalisades, scattered gal-3 ligands were also found in these microenvironments (Figure S1). These ligands could be modulating cell adhesion in pseudopalisading cells, which would be migrating out of necrotic foci. Galectin-3 functions, however, seem first related with tumor cell survival in stressed microenvironments (hypoxic *and* serum-deprived), such as those found in pseudopalisades.

Gal-3 upregulation in hypoxic conditions was suggested by both transcriptomic and proteomic approaches in different *in vitro* models [20]–[22]. Here, we have evaluated the impact of two critical stressing factors of the tumor microenvironment on gal-3 expression, namely hypoxia and serum deprivation. Our results indicate that gal-3 expression is part of a protective cellular response to both conditions, commonly associated with decreased protein synthesis [46]. Current evidence indicated that gal-3 participates in the endocytic process, allowing for the bending of membranes around molecules which will be processed by cells [47].

In one-dimensional electrophoresis, gal-3 protein presents two distinct electrophoretic forms in NG97ht cells and these observations led to the possibility of post-translational modifications, such as phosphorylation [48]–[50]. Particularly, phosphorylation at the Ser^6^ is related to gal-3 anti-apoptotic effect and its export from the nucleus to the cytoplasm [49], [51]. Hill and et al. [52] had shown that tyrosine- phosphorylation of gal-3 was upregulated in rat colon carcinogenesis, whereas serine-phosphorylation of gal-3 was downregulated. Our results demonstrate that phosphorylation is a common posttranslational modification of gal-3, especially observed in its most acidic isoforms in normoxia. In addition, it seems that the NG97ht cell line expresses two different gal-3 alleles differing in one amino acid. It is not clear what is the functional role of this particular variant found in NG97ht cell line. Other variants, such as the ^292^Arg and ^292^Cys variant seem clinically relevant, as discussed elsewhere [53]. Our initial hypothesis was that a posttranslational modification could lead to accumulation of gal-3 in cells exposed to hypoxia. We found no evidence to back up this idea in the experiments performed herein. Previous data from Cao's group had suggested that tyrosine-phosphorylation of gal-3 would prevent its degradation by the autophagic system [54].

In different cell types, gal-3 was clearly upregulated under hypoxia through HIF-1α dependent pathways [21], [44]. Our results indicate not only the participation of HIF-1α, but also NF-κB, which was also upregulated *in vivo* in pseudopalisades. The NF-κB family of transcription factors contribute to the regulation of the HIF-1α gene [55], [56] and it has been shown that NF-κB is also induced upon hypoxia [57], [58], contributing for gal-3 induction and protection against cell death under oxygen and nutrient deprivation. Nonetheless, other studies demonstrated the importance of other transcriptional factor in gal-3 induction, such as members of the RUNX family [59]–[62].

However, gal-3 induction under CoCl_2_ seems to present some peculiarities in comparison to hypoxia: (i) it was more sensitive to NF-κB inhibition; and, (ii) it did not present changes with the HIF-1αinhibitor, although HIF-1α accumulated in the cells' nuclei, which suggests that HIF-1α may play a minor role in gal-3 induction with CoCl_2_. Indeed, Olbryt and colleagues [20] had shown that the groups of genes whose expression were modified under either CoCl_2_ or low pO_2_ shared similarities, but were not identical. Also, heavy metal salts such as NiCl_2_ and CoCl_2_ may activate NF-κB [63] through the induction of the translocation of NF-κB p65 and also p50 into the nucleus and may enhance NF-κB DNA binding activity.

Hypoxic conditions induce different types of cell death, including apoptosis, as previously demonstrated in a neuroblastoma cell line [64] and also in the exposure to CoCl_2_ in glioblastoma cells [65]. Our results suggest a relationship between nutrient deprivation, caspase activation, hypoxia and ROS increase in the induction of cell death in the cells studied herein, which could be induced by the decoupling of the mitochondrial respiratory chain [66]. Hypoxia, however, was not sufficient to induce cell death, since cells needed also to be nutrient-deprived, mimicking the conditions of complete environmental stress observed in tissues distant apart from functional vessels, which lack both oxygen and nutrient distribution. Cell death induced by CoCl_2_, regardless nutrient availability, could be due to the heavy metal cytotoxicity.

Under other conditions, however, cell death induction followed distinct paths, as demonstrated by Steinbach and colleagues [67], who analyzed different glioma cell lines, LN-229, U87MG and LN-18, exposed to oxygen and glucose (and not whole nutrient) deprivation, where cells underwent necrosis, not apoptosis. For CoCl_2_, it has also been indicated its involvement in cell necrosis, but not apoptosis [68]. Overall, studies seem to demonstrate a different array of types of cell death regarding hypoxia.

The NF-kB inhibitor DHMEQ increased death of nutrient-deprived cells exposed to both normoxia and hypoxia, but not to CoCl._2_. Gal-3 expression was downregulated by DHMEQ, which was also shown to decrease the expression of other tumor survival factors in classical Hodgkin lymphomas and induce ROS [69].

The increase of gal-3 under exposure to hypoxic conditions and serum starvation suggests two distinct scenarios. In a first scenario, gal-3 could be one of the inducers or sensitizers of cell death. This scenario is usually dependent on the secretion of gal-3 and its deposition in the extracellular matrix. We had shown that upon infection with *Trypanosoma cruzi*, the causative agent of Chagas' disease, there was an increased deposition of gal-3 in the thymic extracellular matrix. Gal-3 increased deposition led to either increased migration of thymocytes out of the thymus or to increased cell death, leading to thymic atrophy [70]. Accordingly, interaction of extracellular gal-3 with undersialylated β1 integrins from colon carcinoma cells triggered apoptosis [18]. However, our results indicate minor participation of extracellular gal-3 in the induction of cell death (Figure S4). In a second scenario, gal-3 could be part of a pro-survival response triggered by hypoxia and serum deprivation. The pro-survival role of gal-3 is often associated with its compartmentalization to mitochondria, where gal-3 maintains the homeostasis of this death-decision organelle [71], [72]. It seems more likely that the results presented here corroborate with this scenario. Using siRNA/shRNA strategies, we could achieve a reduction of the gal-3 protein level and we could also sensitize NG97ht cells to death, thus *de novo* synthesized gal-3 plays an anti-apoptotic role in cells exposed to hypoxia and serum deprivation. Experiments with the human T98G cell line also demonstrated that the protection granted by gal-3 under hypoxia and nutrient deprivation, indicating this protective role of gal-3 is a more general process.

One of the hypothesis is that gal-3 would act as a homeostatic factor in the mitochondrial membrane [12], [13], [71], [73], but also there seems to be a role for gal-3 in the protection against both reactive oxygen and nitrogen species generated in the context of ischemia and reperfusion [7], and in the maintenance of an anti-oxidant intracellular environment [19]. In a model of malignant transformation of melanocytes into melanoma cells, loss of gal-3 was associated with decreased expression of glutathione-S-transferase and several other antioxidant enzymes, leading to a pro-oxidant cellular environment [74]. Another interesting possibility was raised by the findings that gal-3 interacts directly with F(1)F(0)-ATP synthase [75]. Recent evidence using tandem affinity purification and mass spectrometry show gal-3 interacting with mitochondrial proteins such as ATP5C1 and PDHX [76].

Gal-3 seems to be a key factor in tumor engraftment and growth, as we showed here. Similar results have been shown by other researchers in pancreatic cancer cells, for example [77]. However, opposite results were found in a prostate cancer cell line, LNcaP, which does not express gal-3 [78]. This decreased gal-3 expression *in vivo* may also modulate the tumor immune response, especially the interaction with macrophages. Dumont and colleagues [79] have demonstrated that the supernatant from M1 polarized macrophages, which produce TNF-α, IL-1β and ROS, can induce tumor cell growth inhibition *in vitro*, and in gal-3 knockdown cells, it can increase cell death susceptibility. Noteworthy, when cells devoid of gal-3 are engrafted in gal-3 null mice, there is a significant delay on tumor growth [80]. Here we have silenced gal-3 in human glioma cells and implanted the engineered cells subcutaneously. Tumors grew at a very slow pace and were very heterogeneous regarding gal-3 expression; small tumors tended to be positive for gal-3, while larger tumors were gal-3 negative, but accumulated gal-3 in the pseudopalisading cells. As demonstrated, gal-3 expression is dynamic and it seems especially important in early phases of tumor engraftment and in selected microenvironments.

Gal-3 may play other roles in cells from hypoxic and serum deprived areas, since gal-3 could also elicit cell migration and invasion [81], [82]. Altogether, gal-3 expression would determine the fitness of cells exposed to these stressing microenvironments. Intracellular levels would favor cell survival; upon secretion, gal-3 could act either as an autocrine or a paracrine motility factor, favoring cellular migration out of ill perfused tissue environments. Gal-3 could also lead to angiogenesis [3], [5], [6], which in turn would restore homeostasis of hypoxic and nutrient deprived tissue microenvironments.

In conclusion, based on these *in vitro* and *in vivo* results, we suggest that gal-3 accumulation is part of an adaptive program leading to tumor cell survival in hypoxic *and* nutrient-deprived tumor microenvironments. Therefore, gal-3 is a potential target for sensitizing glioma cells to death.

## Supporting Information

Figure S1 **Detection of gal-3 ligands in tumor pseudopalisades.** NG97ht cells were inoculated in the flank of nude mice and when tumors were fully grown, they were formalin fixed and paraffin embedded for the analysis of galectin-3 ligands. A modified gal-3, which was fused with an alkaline phosphatase enzyme, was used in this process and results demonstrated the availability of gal-3 ligands in pseudopalisading areas (red staining) around necrotic areas (*). Top right figure is the negative control. Scale bar 40 µm.
(TIF)

Figure S2 **Detection of hypoxia induction** ***in vitro*** **by** **pimonidazole**. NG97ht cells were treated with pimonidazole and exposed to normoxia, CoCl2 100 µM and hypoxia in deprived medium for 2 h. A. Cells exposed to normoxia (***A***) did not demonstrate pimonidazole adducts, which are only formed in hypoxia under 14 µM of oxygen, or less than 10 mmHg (***C***). ***B***. Also, pimonidazole adducts were identified in cells exposed to CoCl2 100µM. Cells nuclei were stained with DAPI, (***D***) normoxia, (***E***) CoCl2 100 µM, (***F***) hypoxia (original magnification, 200×).
(TIF)

Figure S3 **Gal-3 siRNA treatment increases the rate of non-proliferating cells.** Analysis of the G0-G1/S-G2-M proportion (rate of non-proliferating cells) in NG97ht cells treated with gal-3 siRNA or scramble siRNA and exposed to either normoxia, CoCl2 100 µM or hypoxia in complete or deprived medium. Cells transfected with the gal-3 siRNA demonstrated an increase in the proportion of non-proliferating cells compared to cells transfected with scramble siRNA in normoxia in complete medium, and hypoxia, both in complete or serum-deprived medium. Representative experiment of at least two independent assays and data are presented as mean±SEM using Two-Way ANOVA. * p<0.05; *** p<0.001.
(TIF)

Figure S4 **Extracellular gal-3 plays a minor role in the cell death induction in hypoxia.** NG97ht cells were exposed to normoxia, CoCl_2_ 100 µM and hypoxia in serum-deprived medium for 48 h and they were also incubated with a gal-3 binding carbohydrate, Galβ1-4GlcNacβ-Sp 200 µM, a control carbohydrate, sucrose 200 µM, or no carbohydrate. Hypodiploid cells were then counted by propidium iodide staining using a flow cytometry. Results demonstrated a decrease in cell death in cells exposed to Galβ1-4GlcNacβ-Sp in comparison to sucrose. Representative experiment of at least three independent assays and data are presented as mean±SEM using Two-Way ANOVA. * p<0.05.
(TIF)

Figure S5 **Gal-3 and GFAP analysis in gal-3 and scramble shRNA U87MG tumor derived cells**. U87MG cells were transduced with an shRNA sequence for gal-3 silencing and a control scramble shRNA and then cells were inoculated in the flank of nude mice. After 33 days, scramble shRNA cells had grown and demonstrated necrosis (*) with pseudopalisading cells (arrow), which were positive for gal-3 (***A***). Gal-3 shRNA derived tumors developed more slowly and reached a grown size after 64 days, demonstrating also necrosis (*) with pseudopalisading (arrow) positive for gal-3 (***B***). The analysis of the gal-3 shRNA tumor derived cells after 33 days post inoculation revealed that these tumors upregulate and express gal-3 throughout the cells (***C***). GFAP staining demonstrated the glial origin of these cells (***D-E-F***). HE staining (***G-H-I***). Scale bar 200 µM.
(TIF)
