# Supplementary figures and images for: Galectin-3 Up-Regulation in Hypoxic and Nutrient Deprived Microenvironments Promotes Cell Survival
Source: PLoS One. 2014 Nov 4;9(11):e111592. doi: 10.1371/journal.pone.0111592 (PMC4219723; doi:10.1371/journal.pone.0111592)

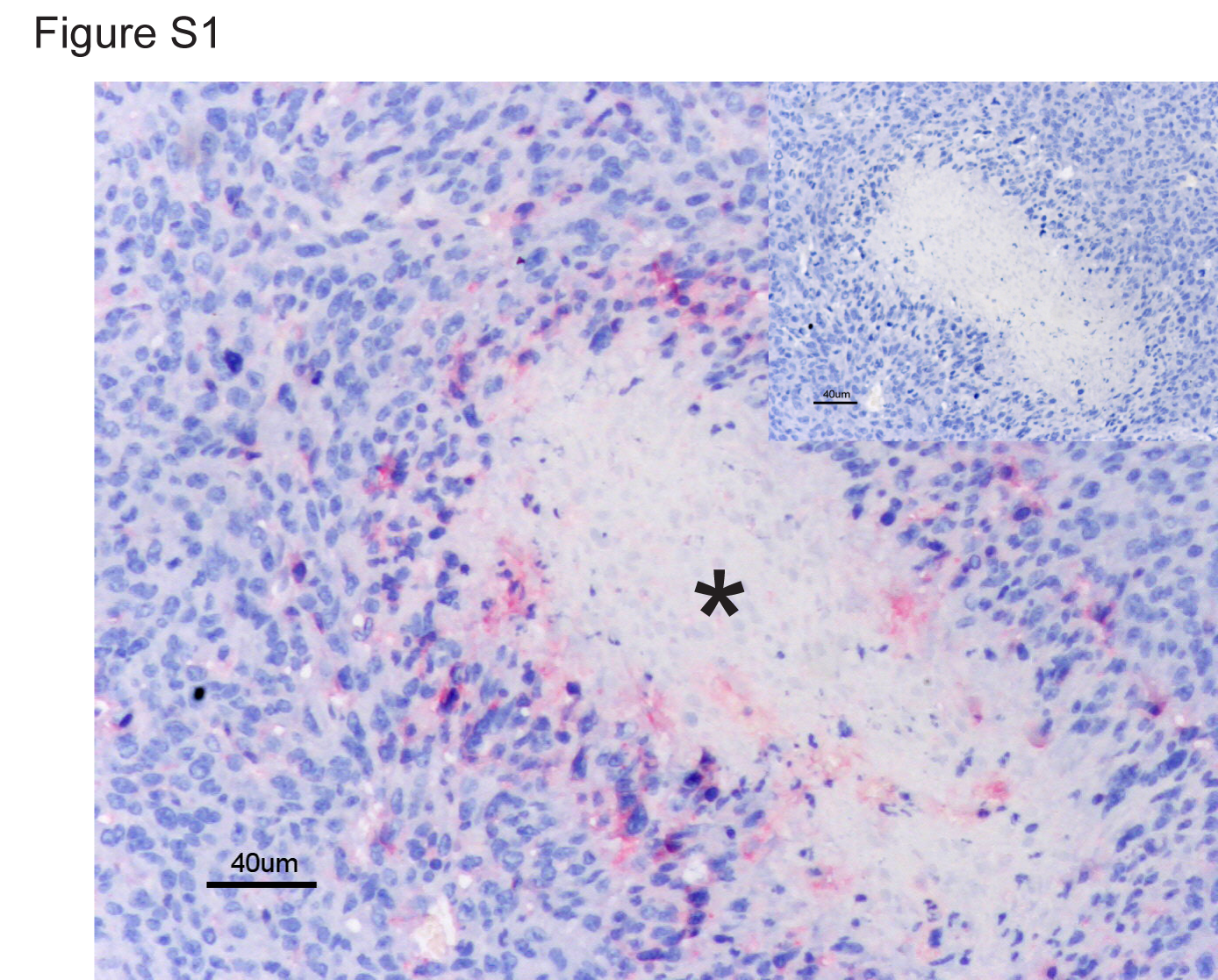

Supplement: Figure S1 — Detection of gal-3 ligands in tumor pseudopalisades. NG97ht cells were inoculated in the flank of nude mice and when tumors were fully grown, they were formalin fixed and paraffin embedded for the analysis of galectin-3 ligands. A modified gal-3, which was fused with an alkaline phosphatase enzyme, was used in this process and results demonstrated the availability of gal-3 ligands in pseudopalisading areas (red staining) around necrotic areas (*). Top right figure is the negative control. Scale bar 40 µm. (TIF) [file pone.0111592.s001.tif]

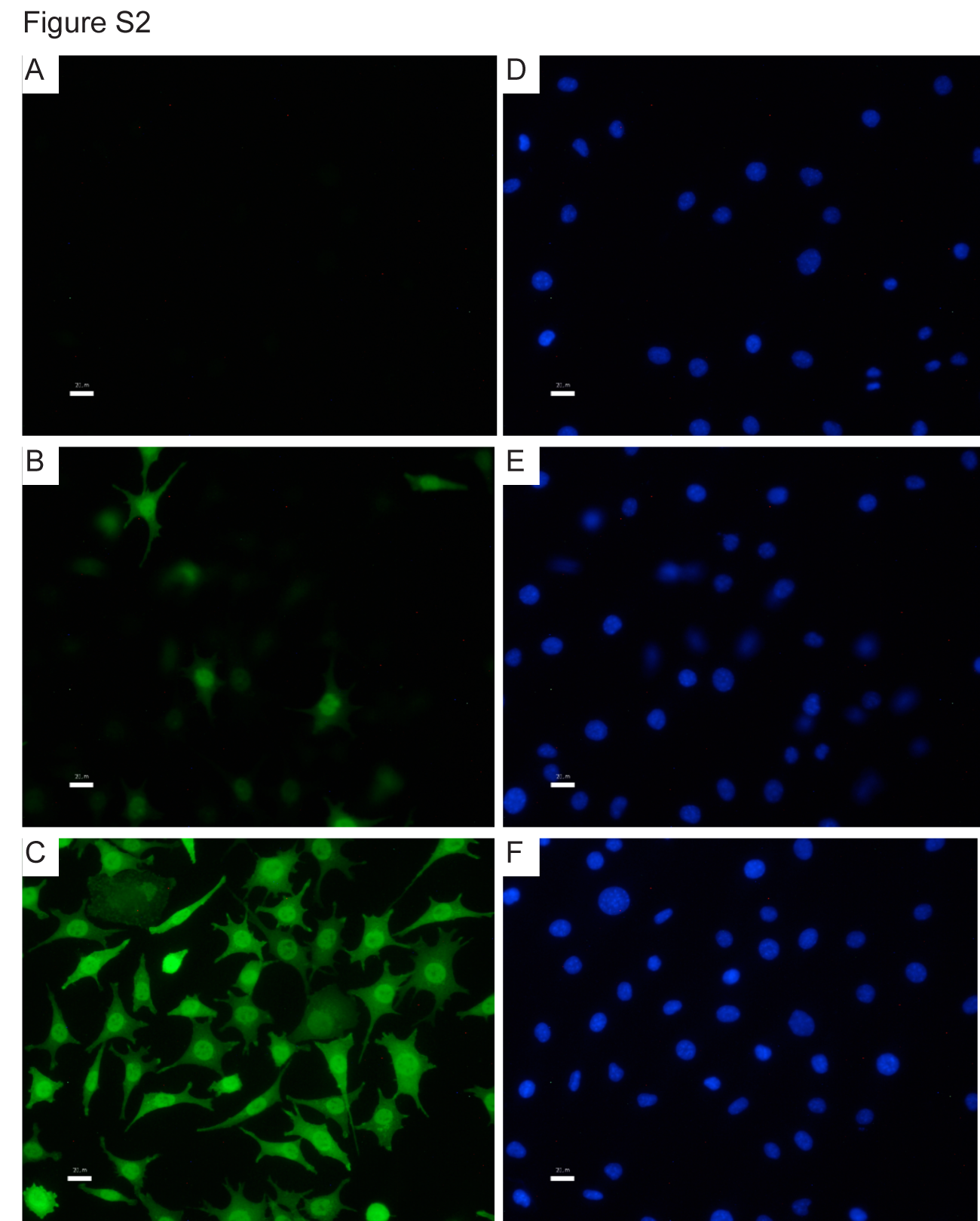

Supplement: Figure S2 — Detection of hypoxia induction in vitro by pimonidazole. NG97ht cells were treated with pimonidazole and exposed to normoxia, CoCl2 100 µM and hypoxia in deprived medium for 2 h. A. Cells exposed to normoxia (A) did not demonstrate pimonidazole adducts, which are only formed in hypoxia under 14 µM of oxygen, or less than 10 mmHg (C). B. Also, pimonidazole adducts were identified in cells exposed to CoCl2 100µM. Cells nuclei were stained with DAPI, (D) normoxia, (E) CoCl2 100 µM, (F) hypoxia (original magnification, 200×). (TIF) [file pone.0111592.s002.tif]

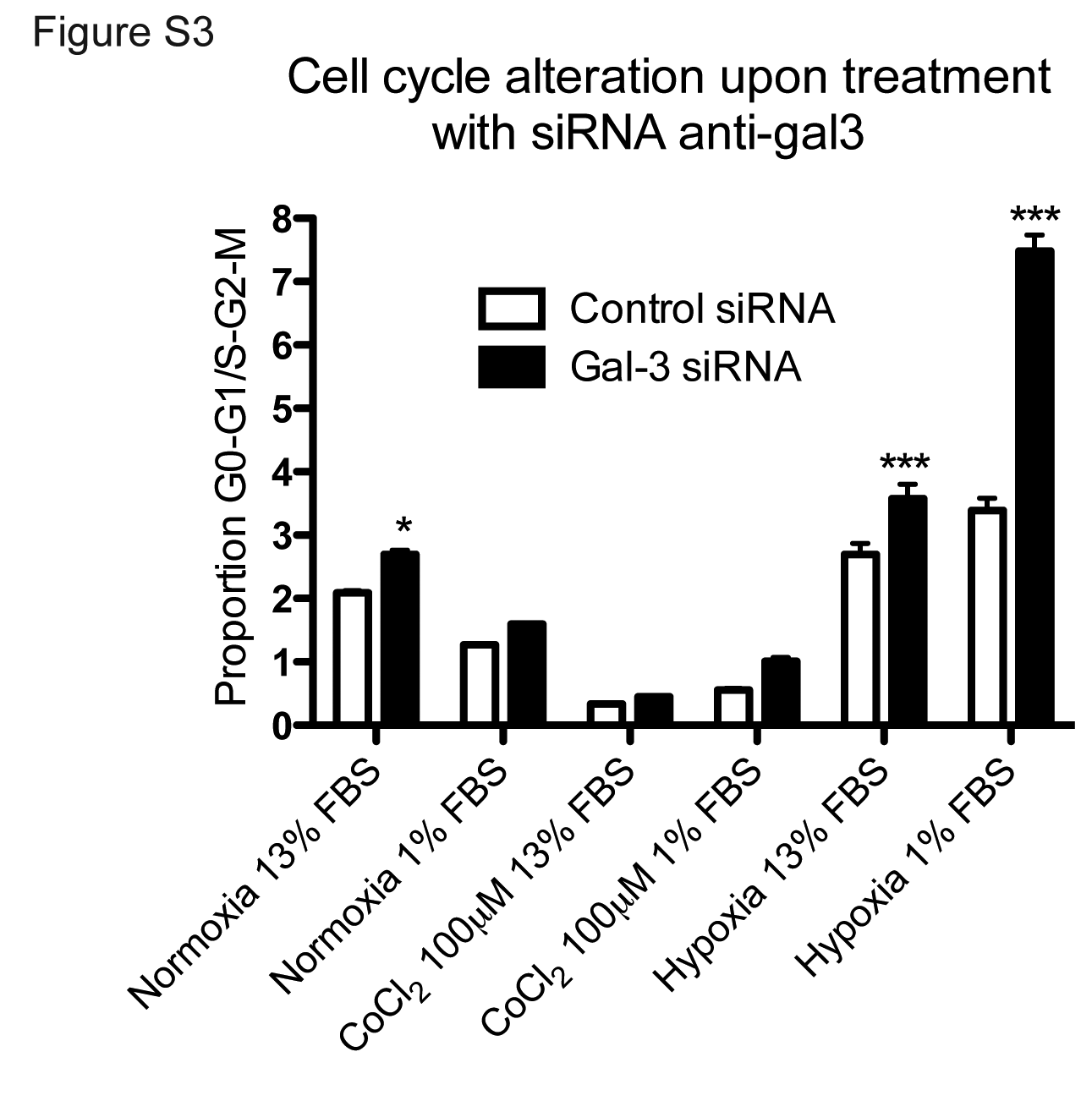

Supplement: Figure S3 — Gal-3 siRNA treatment increases the rate of non-proliferating cells. Analysis of the G0-G1/S-G2-M proportion (rate of non-proliferating cells) in NG97ht cells treated with gal-3 siRNA or scramble siRNA and exposed to either normoxia, CoCl2 100 µM or hypoxia in complete or deprived medium. Cells transfected with the gal-3 siRNA demonstrated an increase in the proportion of non-proliferating cells compared to cells transfected with scramble siRNA in normoxia in complete medium, and hypoxia, both in complete or serum-deprived medium. Representative experiment of at least two independent assays and data are presented as mean±SEM using Two-Way ANOVA. * p<0.05; *** p<0.001. (TIF) [file pone.0111592.s003.tif]

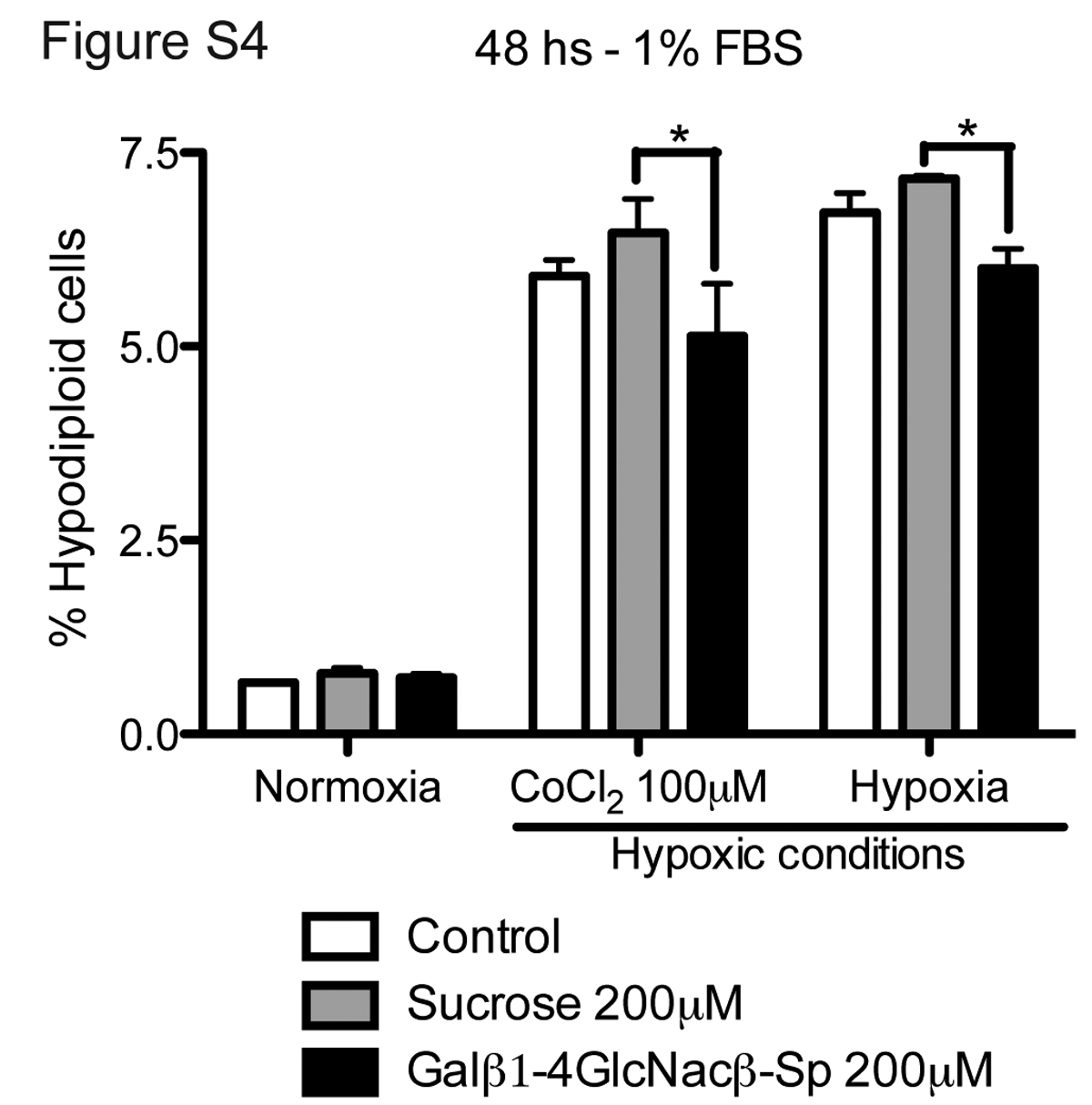

Supplement: Figure S4 — Extracellular gal-3 plays a minor role in the cell death induction in hypoxia. NG97ht cells were exposed to normoxia, CoCl2 100 µM and hypoxia in serum-deprived medium for 48 h and they were also incubated with a gal-3 binding carbohydrate, Galβ1-4GlcNacβ-Sp 200 µM, a control carbohydrate, sucrose 200 µM, or no carbohydrate. Hypodiploid cells were then counted by propidium iodide staining using a flow cytometry. Results demonstrated a decrease in cell death in cells exposed to Galβ1-4GlcNacβ-Sp in comparison to sucrose. Representative experiment of at least three independent assays and data are presented as mean±SEM using Two-Way ANOVA. * p<0.05. (TIF) [file pone.0111592.s004.tif]

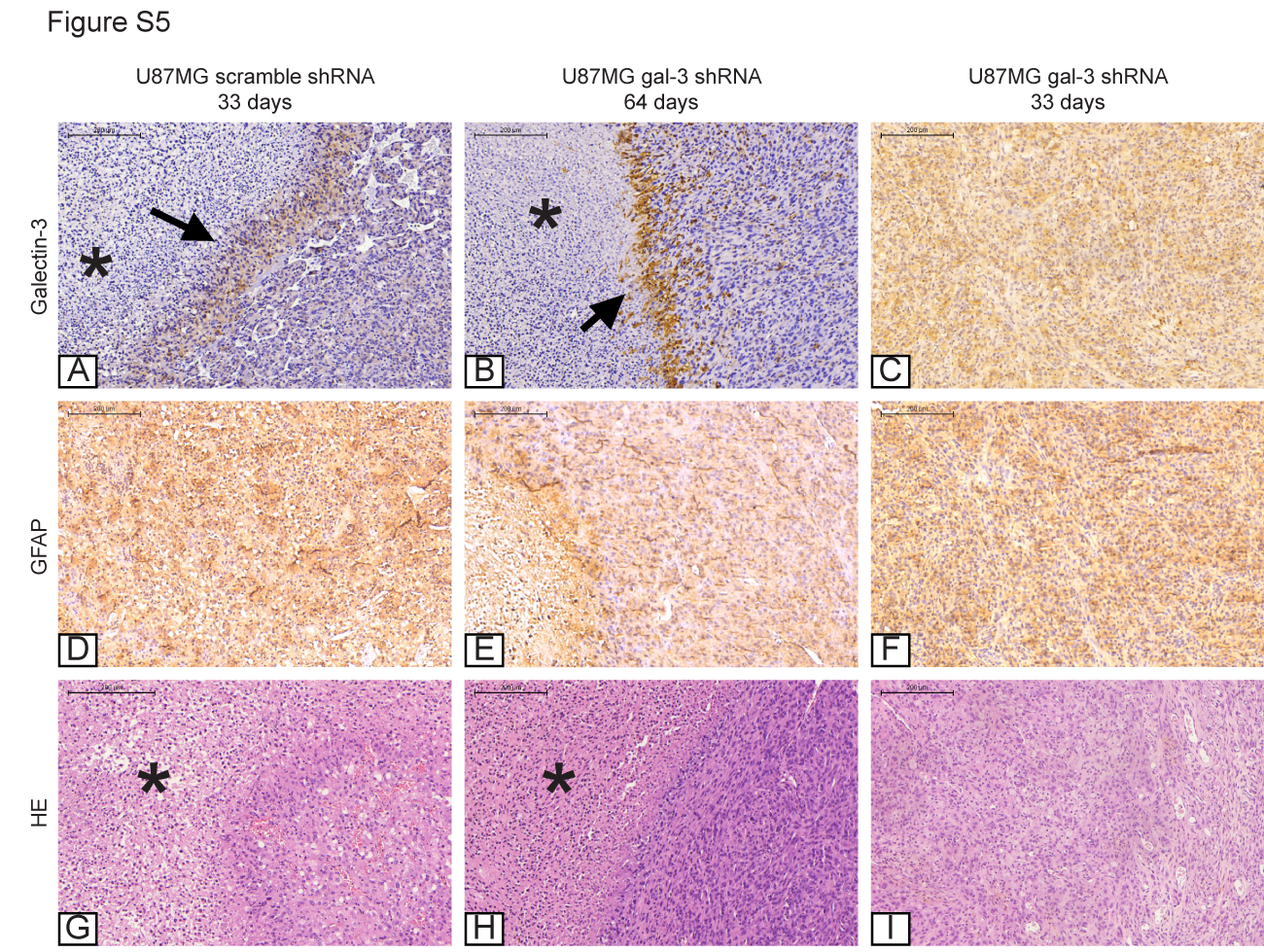

Supplement: Figure S5 — Gal-3 and GFAP analysis in gal-3 and scramble shRNA U87MG tumor derived cells. U87MG cells were transduced with an shRNA sequence for gal-3 silencing and a control scramble shRNA and then cells were inoculated in the flank of nude mice. After 33 days, scramble shRNA cells had grown and demonstrated necrosis (*) with pseudopalisading cells (arrow), which were positive for gal-3 (A). Gal-3 shRNA derived tumors developed more slowly and reached a grown size after 64 days, demonstrating also necrosis (*) with pseudopalisading (arrow) positive for gal-3 (B). The analysis of the gal-3 shRNA tumor derived cells after 33 days post inoculation revealed that these tumors upregulate and express gal-3 throughout the cells (C). GFAP staining demonstrated the glial origin of these cells (D-E-F). HE staining (G-H-I). Scale bar 200 µM. (TIF) [file pone.0111592.s005.tif]
